# RNA Therapeutics in Heart Failure

**DOI:** 10.1007/s12265-025-10717-9

**Published:** 2025-10-30

**Authors:** Frederik E. Deiman, Myrthe M. de Graaf, Herman H. W. Sillje, Niels Grote Beverborg, Nils Bomer, Peter van der Meer

**Affiliations:** Department of Cardiology, University Medical Center Groningen, University of Groningen, Groningen, The Netherlands

**Keywords:** Heart failure, RNAi, RNA therapeutics, Antisense oligonucleotides, siRNA, miRNA, Calcium handling, Myocardial fibrosis, Oxidative stress, Inflammation

## Abstract

RNA-based therapeutics, such as small interfering RNAs (siRNAs), antisense oligonucleotides (ASOs) and messenger RNAs (mRNAs) are promising therapeutics that offer new avenues for targeting molecular pathways underlying heart failure (HF) pathogenesis. This review provides an overview of RNA therapeutics, detailing their mechanisms and potential applications in the treatment of HF. Key pathological processes in HF, including dysregulated calcium handling, myocardial fibrosis, oxidative stress, inflammation and aberrant signalling, are explored to identify how RNA-based therapeutics can be utilized to address these mechanisms. Preclinical studies demonstrating the potential of RNA therapeutics to modulate these pathways are discussed. In addition, the review identifies novel therapeutic targets of HF that may allow more precise and effective interventions, potentially reversing disease progression in HF. In this way, the potential of RNA therapeutics as a next-generation treatment strategy for HF are highlighted, offering hope for more targeted and personalized approaches for HF.

**Graphical Abstract:**

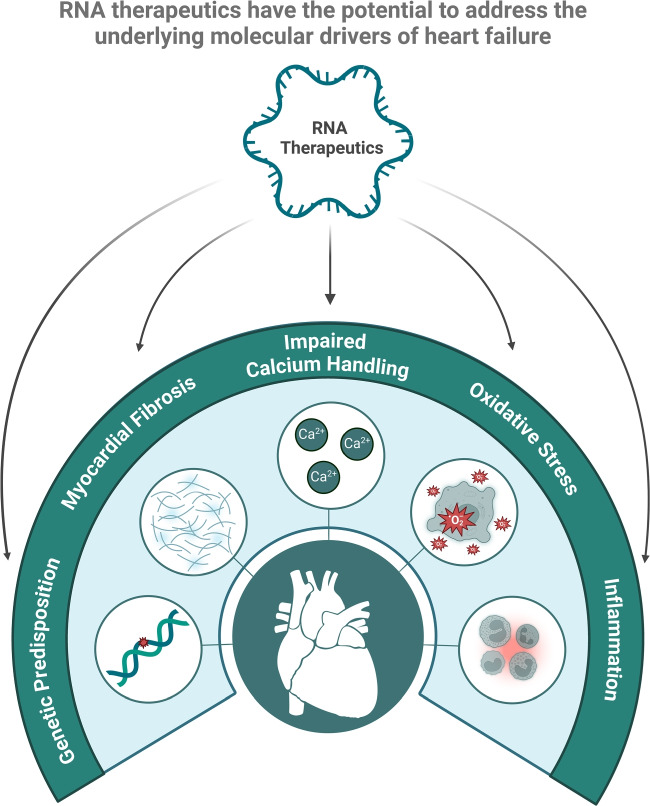

## Introduction

**Fig. 1:**
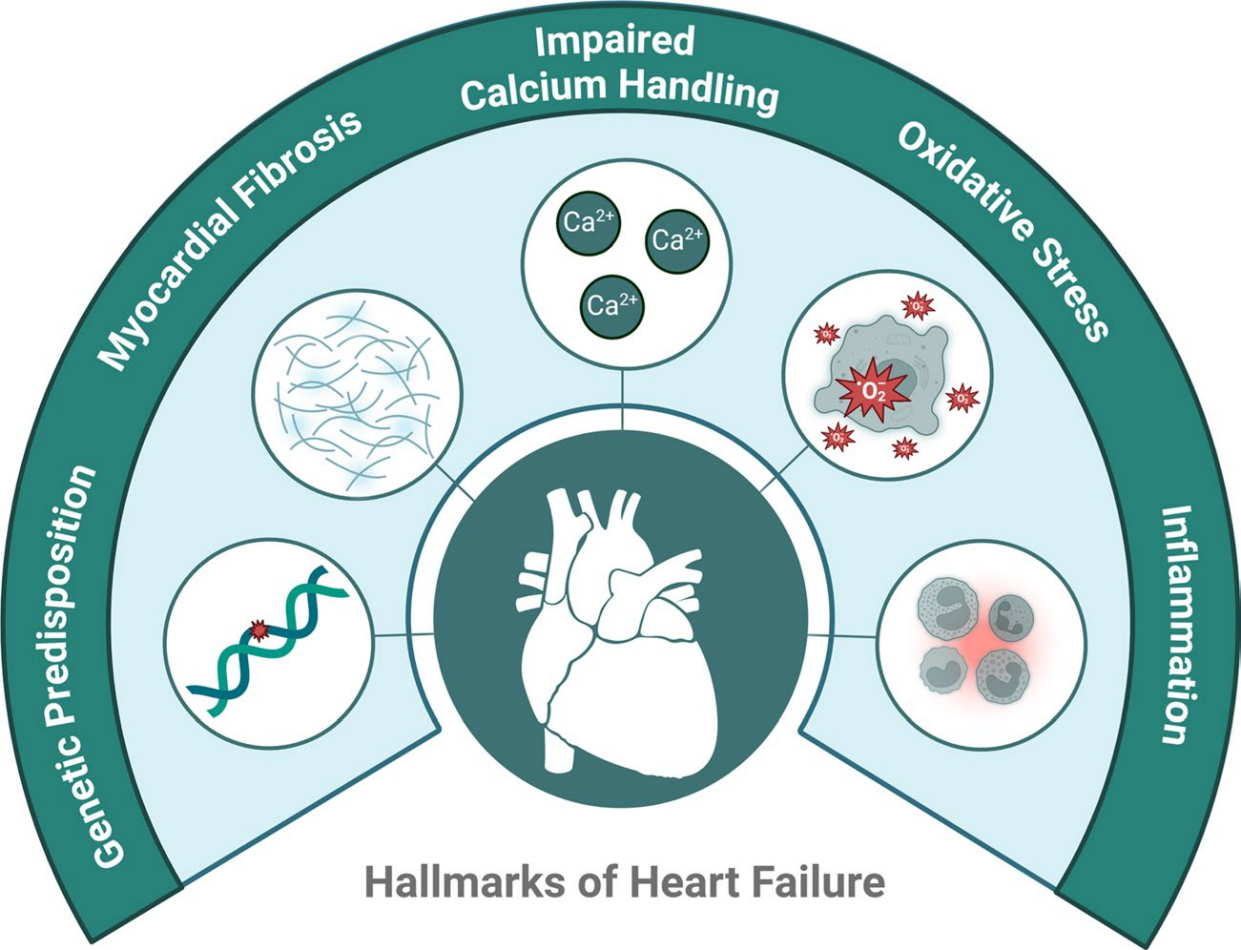
**Therapeutic targets for HF using RNA therapeutics.** Ca^2+^ handling, myocardial fibrosis, oxidative stress, inflammation and genetic predisposition are key pathological processes in HF that are not addressed using traditional HF therapies

Heart failure (HF) is a leading cause of morbidity and mortality worldwide, affecting over 64 million people globally. It represents a significant burden on healthcare systems due to frequent hospitalizations and high treatment costs [1]. The disease is characterized by the heart’s inability to pump blood effectively, leading to symptoms such as shortness of breath, fatigue, and fluid retention. Key pathological mechanisms driving HF include impaired calcium (Ca^2+^) handling, myocardial fibrosis, oxidative stress, and inflammation, all of which contribute to progressive cardiac dysfunction (Fig. 1) [2]. Traditional therapies focus on alleviating symptoms, neurohormonal signalling and slowing disease progression, but they often fall short in addressing the underlying molecular abnormalities [3].

RNA therapeutics have recently emerged as a powerful new class of treatment [4], with several comprehensive reviews highlighting advances across a wide range of cardiovascular diseases [5–10]. Their key advantage lies in their ability to directly target disease-associated transcripts, offering greater specificity, flexibility, and the potential to modulate genes and pathways that could previously not be targeted. Unlike conventional drugs that typically act on proteins, RNA-based therapies can intervene earlier in the gene expression cascade, providing a mechanism for both silencing pathogenic genes and replacing deficient ones [4, 11]. However, to date, RNA therapeutics have not been implemented in the clinical management of HF, despite their potential to specifically target key molecular mechanisms underlying the condition. The complexity of HF underscores the need for targeted therapies, and RNA therapeutics offer a promising means to address underlying disease mechanisms beyond what conventional treatments can achieve.

In this review, we aim to bridge this gap by providing a focused overview of RNA therapeutics with potential in HF. Unlike broader cardiovascular reviews, we specifically focus on HF pathophysiology and explore how RNA therapeutics such as small interfering RNAs (siRNAs), antisense oligonucleotides (ASOs), and mRNA therapies can be harnessed to modulate disease-driving pathways. We also examine both preclinical and emerging clinical evidence to evaluate the translational potential of RNA therapeutics in HF.

## RNA Therapeutics

### RNA Interference Strategies

**Fig. 2:**
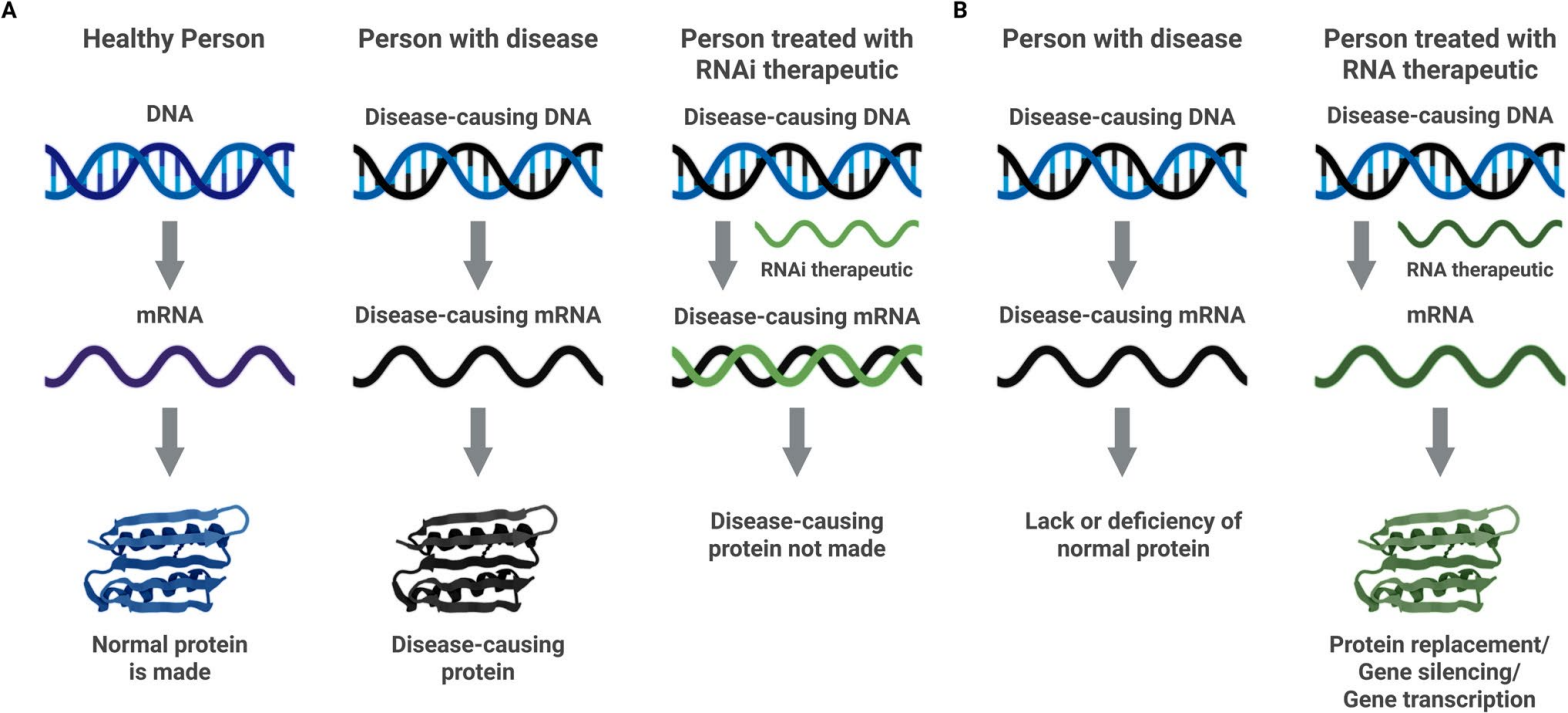
**The principle of RNA therapeutics.** **A** RNA interference, In the central dogma of biology mRNA is translated into protein. However, in the context of disease, a disease-causing protein is present. RNAi therapeutics inhibit gene expression by degrading target mRNA or preventing its translation. **B** RNA enhancing therapeutics, RNA therapeutics can be utilized to allow protein replacement, gene silencing or gene transcription. In this way, patients lacking or deficient in a protein can receive mRNA that encodes missing protein or silences harmful biological pathways

**Fig. 3:**
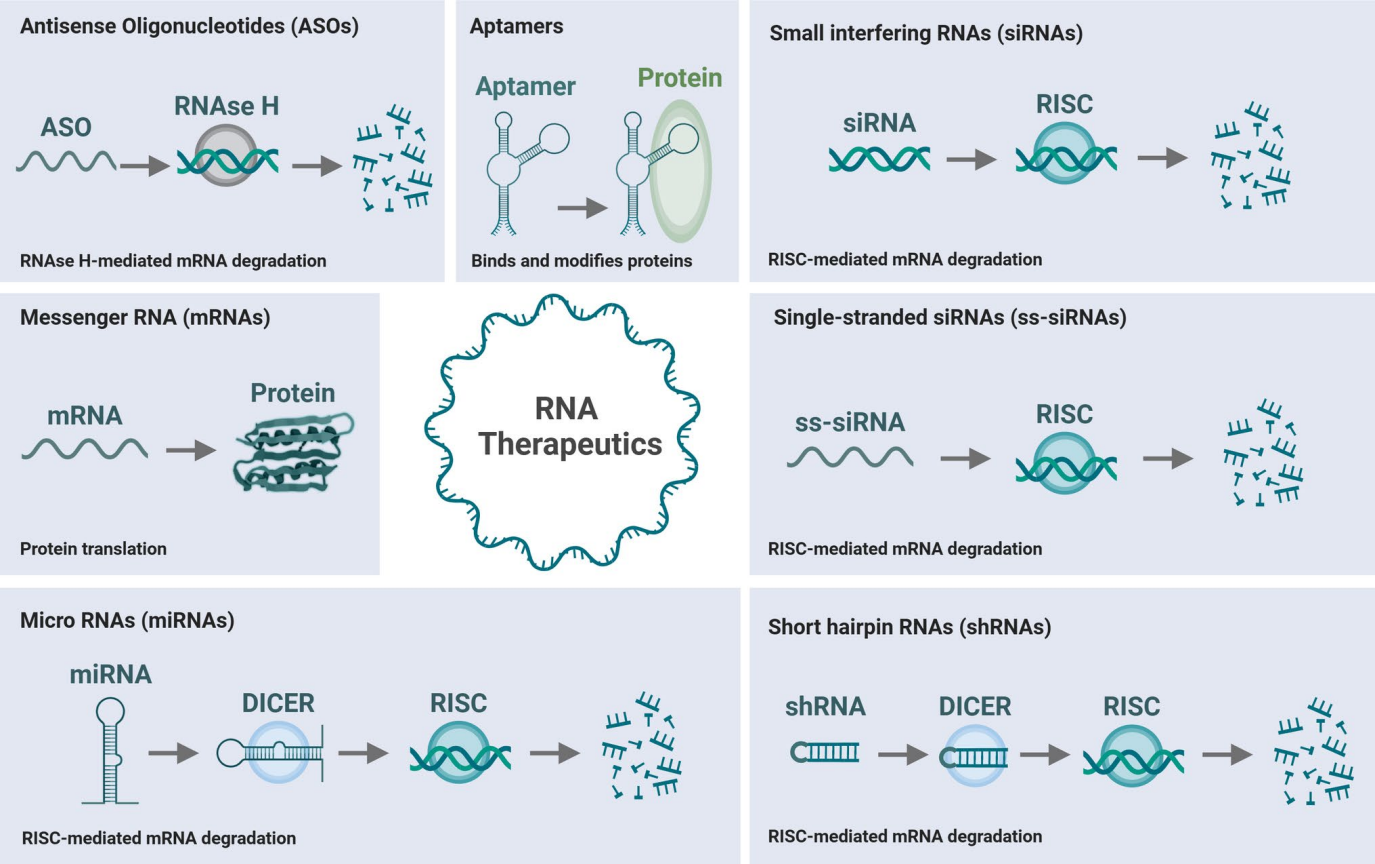
**Overview of RNA therapeutics.** RNA therapeutics can interfere with or enhance mRNA. ASOs utilize RNAse H-mediated mRNA degradation. siRNAs, ss-siRNAs, shRNAs and miRNAs utilize RISC-mediated mRNA degradation. Aptamers allow blockage of pathological interactions. In contrast, mRNAs can be delivered directly into the cell allowing translation of protein

RNA interference (RNAi) is a biological process in which small RNA molecules inhibit gene expression by degrading target mRNA or preventing its translation (Fig. 2A). RNAi plays a key role in gene regulation and is widely used as a tool for gene silencing in research and therapeutic contexts [12]. RNAi strategies can be distinguished by their type, including ASOs [13], siRNA [14], micro RNA (miRNA) [14], short hairpin RNA (shRNA) [15], single-stranded siRNA (ss-siRNA) [16] and aptamers (Fig. 3) [17].

Here, ASOs are single-stranded synthetic oligonucleotides that bind to specific mRNA sequences, blocking their translation or promoting their degradation. ASOs bind to complementary mRNA, forming an RNA–DNA hybrid. This can inhibit translation, induce mRNA degradation via Ribonuclease H (RNAse H; which cleaves RNA in RNA–DNA hybrids), or modulate splicing [13]. ASOs have shown great potential and are clinically used as a therapy for hereditary diseases by modulating the expression of specific genes, such as in the treatment of muscular dystrophy [18] and amyloidosis [19].

siRNAs are double-stranded RNA molecules (~ 21–23 nucleotides) that target specific mRNA for degradation, leading to gene silencing. Exogenously administered or endogenously produced siRNA binds to the RNA-induced silencing complex (RISC). This RISC-siRNA complex then binds to complementary mRNA, which is subsequently cleaved and degraded [14]. It is used for targeted gene knockdown in research and therapies, especially for diseases caused by overexpressed or faulty genes.

miRNAs are small non-coding RNA molecules (~ 20–25 nucleotides) that regulate gene expression by blocking mRNA translation or promoting mRNA degradation, and are related to patient outcomes in HF [20]. miRNAs are processed from precursor forms (pri-miRNA, pre-miRNA) and incorporated into RISC. miRNAs bind to partially complementary sequences on target mRNA, often in the 3' untranslated region, inhibiting translation or leading to mRNA degradation [14]. miRNAs are involved in fine-tuning gene expression and are implicated in various diseases, including cancer and cardiovascular diseases.

Alternatives that are less commonly used include shRNA, ss-siRNA and Aptamers. shRNAs are artificial RNA molecules designed to produce siRNA-like effects but with higher stability, leading to long-term gene silencing. shRNA is expressed from plasmids or viral vectors and forms a hairpin structure. Once processed in the cell, it is converted into siRNA, which then guides RISC to degrade target mRNA [15, 21].

ss-siRNA is a simplified form of siRNA technology, using only a single RNA strand instead of the traditional double-stranded siRNA, offering increased stability and reduced off-target effects. ss-siRNA is chemically modified to maintain stability and prevent degradation in cells. Once introduced into the cell, ss-siRNA is incorporated into the RISC complex. Unlike conventional double-stranded siRNA, ss-siRNA doesn't require strand separation, and it guides RISC to degrade complementary mRNA directly. ss-siRNAs are used in therapeutic settings where a more stable, specific, and less immunogenic RNA interference mechanism is needed, such as in gene therapies for viral infections, cancers, or genetic disorders [16, 22, 23].

Aptamers are RNA molecules engineered to bind to specific targets, such as proteins or cells, similar to antibodies, and have been reviewed in relation to HF [24] Aptamers can act as antagonists, blocking pathological interactions, or be conjugated with drugs for targeted delivery. Pegaptanib, used in age-related macular degeneration, is a prominent example [17, 25].

siRNA, shRNA, miRNA, and ss-siRNA all silence gene expression by degrading target mRNA, with varying stability, structure, and duration of effect. Here, siRNA and ss-siRNA are short-acting, while shRNA offer longer-term silencing. Aptamers block pathological interactions. miRNA and ASOs regulate gene expression differently, with miRNA fine-tuning multiple genes by inhibiting translation, and ASOs binding mRNA to either block or degrade it. Each of these RNAi mechanisms can be harnessed for therapeutic purposes to selectively target disease-associated genes, particularly in conditions like cardiovascular disease such as HF.

### RNA Enhancing Strategies

In contrast to RNAi strategies, RNA therapeutics can also enhance the expression of specific proteins. These therapeutics primarily involve delivering synthetic messenger RNA (mRNA) directly into the cells (Fig. 2B) [26].

Synthetic mRNA is designed to encode a specific protein of interest, which can then be translated in the cell (Fig. 3). This strategy has been widely recognized through COVID-19 vaccines, which deliver mRNA encoding viral spike proteins to stimulate immune responses. Beyond vaccines, mRNA therapeutics are being explored for protein replacement therapies, where patients lacking or deficient in a protein can receive mRNA that encodes the missing protein. This approach is promising for conditions like enzyme deficiencies and certain metabolic disorders [27, 28].

Other RNA enhancing strategies include small activating RNAs (saRNAs) [29], long non-coding RNAs (as modulators; lncRNAs) [30], circular RNA (circRNA) [31]. However, these strategies are still in early stages of research. saRNAs enhance gene expression of specific genes by binding small RNA molecules to promotor regions or other regulatory elements [29]. lncRNAs influence gene expression at the (post)transcriptional level and have been reviewed in relation to HF extensively [30, 32, 33]. circRNAs are like mRNAs, but highly stable and resistant to degradation, leading to prolonged expression [31].

### Cardiac Delivery of RNA Therapeutics

Cardiac delivery remains a significant challenge in delivery and efficacy of RNA therapeutics in HF and has been extensively reviewed [11, 34–37]. Unlike the liver, where lipid nanoparticles (LNPs) enable efficient and targeted delivery, achieving similar specificity and uptake in the heart is considerably more difficult. Systemic biodistribution, vascular barriers, and low transfection efficiency in cardiomyocytes contribute to poor therapeutic penetration of cardiac tissue [11, 34, 36, 38].

Current delivery strategies include LNPs, adeno-associated viruses (AAVs), and emerging platforms such as exosomes. However, these vectors often exhibit preferential accumulation in non-cardiac organs such as the liver. In the case of viral vectors, concerns about immunogenicity, limited scalability, and challenges with repeated dosing further complicate their use for chronic cardiac conditions [11, 34, 35].

Moreover, RNA molecules are inherently unstable, susceptible to degradation by nucleases, and possess short half-lives. While chemical modifications can enhance stability and reduce immunogenicity, they do not fully address challenges related to cellular uptake and endosomal escape in cardiomyocytes. High dosing is often required to achieve therapeutic efficacy, which in turn increases the risk of off-target effects and systemic immune activation [11, 34, 35].

Despite encouraging progress in vector engineering, cardiac-targeting ligands, and localized delivery approaches, no universally accepted delivery platform, or gold standard, has emerged so far. These delivery limitations, along with the need for patient selection, remain key obstacles to the broader clinical translation of RNA therapeutics in HF.

### RNA Therapeutics and Patient Selection

Patient selection will play a crucial role in the success of RNA therapeutics for HF. Unlike traditional therapies, RNA therapeutics often rely on specific molecular targets whose expression levels or genetic mutations vary across patients. Stratification based on gene expression profiles, genetic variants (e.g., pathogenic mutations amenable to ASO therapy), or biomarker-guided selection could greatly enhance therapeutic efficacy and minimize off-target effects [39, 40]. In addition, patient-specific factors such as immune responsiveness, hepatic or renal function (which can affect RNA therapeutic clearance) [11], and prior exposure to viral vectors (for example, pre-existing antibodies to AAV capsids) must be considered [34]. As such, RNA therapeutics may be particularly suited for precision medicine in HF, but will require the development of robust diagnostic and stratification tools to identify appropriate candidates.

### Clinical applications of RNA therapeutics in Cardiovascular Disease

The clinical use of RNA therapeutics in cardiovascular disease has been extensively reviewed [5–9]. Although the number of clinical trials is limited, RNA therapeutics have shown great potential in clinical trials for the treatment of cardiovascular disease. Clinical trials are ongoing in cardiac fibrosis [41], hypertension [42], cardiac amyloidosis [43], dyslipidaemia [44], muscular dystrophy [45] and revascularization [46]. These clinical trials have led to major successes that are now approved novel therapies, such as vutrisiran [43] and inotersen [47] for cardiac amyloidosis, eteplirsen [18] for muscular dystrophy and inclisiran [44, 48] for hypercholesterolemia. These novel medicines demonstrate the promise of RNA therapeutics for the treatment of cardiovascular disease, by specifically targeting signal transduction pathways involved in disease pathophysiology. However, RNA therapeutics are not currently being used to treat HF, so there is a window of opportunity. RNA therapeutics can be designed to target key pathological processes in HF, including dysregulated Ca^2+^ handling [49], myocardial fibrosis [50], oxidative stress [51, 52], inflammation [51, 53, 54], and aberrant signalling [55]. As such, RNA therapeutics may represent a promising therapeutic strategy for the treatment of HF in the future.

### Identifying Novel Therapeutic Targets for the Treatment of Heart Failure Utilizing RNA Therapeutics

Key pathological processes in HF include dysregulated Ca^2+^ handling, myocardial fibrosis, oxidative stress, inflammation, and aberrant signalling [2]. These underlying molecular abnormalities are not addressed using traditional HF therapies, which focus on neurohormonal signalling, alleviating symptoms, and slowing disease progression [3]. The molecular mechanisms underlying these pathological processes can be targeted utilizing RNA therapeutics and may lead to more efficient treatment of HF.

### Targeting Calcium Handling

Dysregulation of Ca^2+^ channels are a hallmark of HF. RNA therapeutics can be designed to target genes encoding Ca^2+^ channels that are altered in HF, such as L-type Ca^2+^ channels (Cav1.2) [56] ryanodine receptors (RyR2) [57] or Sodium/Ca^2+^ Exchanger (NCX) [58]. Cav1.2 is a mediator of Ca^2+^ entry into cardiomyocytes, and dysregulation of Cav1.2 is known to contribute to contractile dysfunction in HF [57, 59]. RyR2 is a main regulator of excitation–contraction coupling. Phosphorylation of RyR2 due to abnormal protein kinase A (PKA) and particularly Ca^2+^/calmodulin-dependent protein kinase II (CaMKII) has extensively been implicated in HF pathology and arrhythmias [60]. NCX is the major Ca^2+^ efflux mechanism of cardiomyocytes [61]. In an experimental model, increased NCX activity promoted arrhythmias, exacerbates ischemic necrosis and causes HF [58, 62]. Dysregulation of Cav1.2, RyR2 and NCX are known to contribute to HF [57]. Modulating the expression of these channels utilizing RNA therapeutics can help normalize Ca^2+^ influx and release, potentially improving contractility and reducing arrhythmias.

Ca^2+^ handling is regulated by various kinases and phosphatases including CaMKII [63], PKA [64] and protein phosphatase 1 (PP1). Dysregulation of these kinases has been implicated in HF [63–65]. Interestingly CaMKII and PKA are the kinases responsible for the phosphorylation of Cav1.2 and RyR2 related to HF [65]. CaMKII, via phosphorylation, regulates cardiac excitation–contraction coupling [65]. Upon chronic activation, CaMKII is involved in hypertrophic and inflammatory signaling and promotes apoptosis and arrhythmias [65, 66]. CaMKII is well established to have increased activity in HF, and inhibition of CaMKII attenuates HF [66]. PKA is a key player in cardiomyocyte function and is involved in the regulation of contraction, metabolism, ion fluxes, and gene transcription [67]. Altered PKA activity has been associated with HF by leading to dysregulation of Ca^2+^ fluxes and Ca^2+^ utilization [64, 68]. PKA inhibition is described as a potential target for HF based on data derived from *in vitro* systems [67]. PP1 is another regulator of cardiac excitation–contraction coupling, by exerting effects on cardiac ion channels and Ca^2+^ handling proteins, and dysregulation of PP1 is associated to HF [69]. CaMKII, PKA and PP1 are key regulators of heart function in health and disease. Due to the heavy involvement of CaMKII, PKA and PP1 in HF pathophysiology, these are interesting targets for RNA therapeutics. Clinical gene therapy trials are already ongoing targeting PP1 (ClinicalTrials.gov ID; NCT05598333) and CaMKII (ClinicalTrials.gov ID; NCT02557217), the outcome of these trials will determine the potential of modulating phosphatases and kinases in HF.

Targeting proteins involved in Ca^2+^ uptake and storage, such as sarcoplasmic reticulum Ca^2+^ ATPase (SERCA2a) and phospholamban (PLN) are another way to target Ca^2+^ handling in HF. Enhancing SERCA2a expression using RNA therapeutics can improve Ca^2+^ reuptake into the sarcoplasmic reticulum, aiding in relaxation and contractility [70, 71]. In the CUPID 2 trial, however, no clinical benefit was observed in HF patients after SERCA2a gene delivery [72]. Therefore, other Ca^2+^ regulatory proteins, such as PLN and Dwarf open reading frame (DWORF) have gained more attention for the treatment of HF [55, 73]. PLN is the main regulator of SERCA2a and reducing PLN is postulated to improve SERCA2a activity [74]. In several models of HF, PLN downregulation using RNAi leads to improved contractile function [55, 75]. Another SERCA2a activator is DWORF, that functions by displacing PLN from SERCA2a [76]. *In vivo* studies have shown that DWORF overexpression is known to enhance contractility and attenuate HF [77, 78].

Certain miRNAs are known to regulate Ca^2+^ handling proteins, for example, miR-1 [79–81], miR-25 [82] and miR-133 [83–85]. These miRNAs have been implicated in Ca^2+^ handling, cardiac remodeling and HF. Using RNA therapeutics these miRNAs can be inhibited, which could enhance the expression of Ca^2+^ handling proteins, potentially improving Ca^2+^ cycling. miR-1 is a regulator of hypertrophy and downregulated in patients with symptomatic HF [80]. miR-1 replacement therapy improved cardiac parameters, including Ca^2+^ handling, and reduced fibrosis and apoptosis [79]. miR-25 is a miRNA that is known to potently delay Ca^2+^ uptake kinetics in the heart, that is upregulated in HF. Inhibition of miR-25 improves cardiac contractility and attenuates HF [82]. miR-133 is another regulator of hypertrophy, and an established circulating biomarker for HF. In an *in vivo* model, replacement therapy using miR-133 prevented HF progression [83, 84]. Together, modulation of several miRNAs using RNAi or RNA therapeutics attenuated HF, identifying them as promising targets for the treatment of HF.

RNA therapeutics can be designed to enhance the expression of Ca^2+^ sensitizing proteins, such as certain Troponin C types with an enhanced Ca^2+^ affinity [86]. Ca^2+^ sensitizers enhance the cardiac response to Ca^2+^ without modifying intracellular Ca^2+^ levels [87]. To date, overexpression of Ca^2+^ sensitizers has not been explored but may gain popularity in the future.

### Targeting Myocardial Fibrosis

Myocardial fibrosis is characterised by excessive collagen deposition in the extracellular matrix (ECM) [88]. The development of myocardial fibrosis is driven by complex interactions between circulating pro-fibrotic cell types, growth factors, and pro-inflammatory cytokines that mediate the differentiation of cardiac fibroblasts into myofibroblasts [89]. Myofibroblasts are essential for wound contraction and tissue repair, but in the context of fibrosis, these cells persist and are primarily responsible for excessive collagen production [90]. This increased collagen synthesis leads to ECM remodelling, a critical process in the pathogenesis of fibrosis [91]. Therefore, directly targeting myofibroblast formation or modulating genes involved in collagen synthesis and ECM remodelling holds promise as a potential anti-fibrotic strategy to attenuate the progression of HF.

Non-coding RNAs are recognised as key regulators in maintaining the balance between quiescent fibroblasts and their activated myofibroblasts form [92]. MiR-21 has been identified as a potent inducer of fibroblast to myofibroblast transition (FMT) [93–96]. Inhibition of miR-21 subsequently reduced the proliferation and invasion of cardiac fibroblasts *in vivo*. It also upregulated the expression of fibroblast markers such as vimentin and discoidin and downregulated the expression of myofibroblast markers including α-smooth muscle actin and tensin [93]. In contrast, certain miRNAs counteract FMT and preserve the fibroblast phenotype. For example, transfection of miR-200 mimic or overexpression of miR-200b in fibroblasts suppressed myofibroblast differentiation [97, 98]. Similarly, overexpression of miR519d inhibited myofibroblast marker α-smooth muscle actin expression and fibroblast proliferation [99]. Using RNA therapeutics to modulate FMT may provide a valuable treatment strategy to limit myofibroblast accumulation and potentially reduce fibrosis.

An alternative treatment strategy is to target myofibroblast activation, which is regulated by biochemical signals, including growth factors. Tissue growth factor-β (TGF-β) is widely recognised for its strong and well-established role in inducing myofibroblast activation [100]. Connective tissue growth factor (CTGF) is a matricellular protein that acts downstream of TGF-β to regulate fibroblast proliferation and ECM protein synthesis [90, 101]. By activating myofibroblasts, CTGF promotes the induction of collagen synthesis and accumulation [90, 102]. This is further accelerated by CTGF-induced upregulation of TGF-β expression, which in turn stimulates CTGF production, creating a positive feedback loop [103]. This self-reinforcing mechanism contributes to the progressive nature of fibrosis. Targeting CTGF or TGF-β to disrupt this positive feedback loop may be a promising strategy for antifibrotic therapy. For instance, *in vivo* treatment with miR-503 ASO reduced cardiac fibrosis by inhibiting collagen production, which associated to the downregulation of CTGF and TGF-β expression [104]. Alternatively, miR-181c is implicated in HF, where it promotes fibroblast activation by increasing mitochondrial reactive oxygen species (ROS) production and triggering pro-fibrotic signaling pathways, including TGF-β. Inhibiting miR-181c with RNA therapeutics may offer a strategy to limit oxidative stress–driven fibroblast activation in HF [105, 106].

RNA therapeutics can also modulate TGF-β signalling or its components. One potential target is Sirtuin 7 (SIRT7), which enhances TGF-β signalling. Cardiac fibroblasts derived from homozygous SIRT7-deficient mice or fibroblasts treated with SIRT7 siRNA *in vitro* showed reduced activation of TGF-β signalling compared to controls [107]. Additionally, reduced expression of collagen I and collagen III were observed in cardiac fibroblasts following SIRT7 siRNA treatment [99, 107]. Components of the TGF-β signalling pathway, including receptors and kinases, can be regulated by miRNAs. For example, miR-101a modulates TGF-β receptor type 1 (TGF-βR1) and TGF-β-activated kinase 1 (TAB3), leading to downregulation of collagen gene expression and induction of autophagy in cardiac fibroblasts, thereby minimising collagen release [108, 109]. *In vivo* transfection of atrial fibroblasts with either miR-133 or miR-590 decreased collagen production, by targeting TGF-βR1 and TGF-βR2, respectively [110]. The inhibitory effect was reversed by their respective ASOs, while co-transfection with both miR-133 and miR-590 enhanced the inhibitory effects on collagen production [110]. These findings highlight the potential of RNA therapeutics as a strategy to modulate signalling pathways associated with collagen production and potentially reducing fibrosis.

Targeting proteins directly involved in collagen synthesis, such as collagen type l alpha (COL1A1) and COL3A1, is an alternative way of targeting excessive collagen synthesis in myocardial fibrosis. COL1A1 encodes the α1 chain of type l collagen and COL3A1 encodes the α1 chain of type lll collagen. A significant increase in the protein expression of COL1A1 and COL3A1 was observed in fibrotic myocardium *in vivo* [111], suggesting that targeting these proteins could serve as a potential therapeutic strategy to reduce fibrosis. Treatment with miR-214-3p agomir reduced the myocardial expression of COL1A1 and COL3A1 and attenuated myocardial fibrosis in mice [111]. This finding was consistent with another study showing that upregulation of miR-214 reduced the expression of collagen type l and collagen type lll in cardiac fibroblasts and reduced myocardial fibrosis *in vivo* [112]. Alternatively, downregulation of COL1A1 and COL3A1 utilizing RNAi can be applied to reduce protein levels of type l collagen and type lll collagen [112].

Increased collagen deposition leads to extracellular matrix (ECM) remodelling by disrupting cell–matrix interactions, exacerbating pathological processes and ultimately impairing cardiac function [113, 114]. Matrix metalloproteinases (MMPs) are key mediators of tissue remodelling, as they degrade ECM protein components [115]. While MMP expression is typically low in healthy cardiac tissue, it is upregulated in pathologically diseased tissue undergoing repair and remodelling [116]. In addition, the levels of endogenous tissue inhibitors of matrix metalloproteinases (TIMPs) are lower in the hearts of transplant patients, which is thought to play a role in the increased activity of cardiac MMPs [117]. Therefore, decreasing MMP expression and increasing TIMPs expression in cardiac tissue may be a promising therapeutic target. Silencing of MMP2 in rat cardiomyocytes using siRNA resulted in a decrease in MMP2 protein levels, which in turn led to a decrease in myosin light chain 1 and 2 degradation and improved the contractility of cardiomyocytes exposed to ischaemia–reperfusion injury [118]. MMP2 expression can also be reduced by increasing TIMP4 expression. Both transfection of an adenoviral construct of human TIMP4 into mouse myocardium and transgenic mice overexpressing myocardial TIMP4 showed reduced active MMP2 expression and reduced MMP9 expression. In addition, both strategies increased left ventricular ejection fraction (LVEF) and reduced LV dilation [119]. RNA therapeutics can modulate ECM remodelling, offering an anti-fibrotic treatment strategy, with the potential to attenuate the progression of HF.

Several clinical trials are currently evaluating RNA-based anti-fibrotic therapies. CDR132L, an ASO targeting miR-132, is in phase II of clinical testing (ClinicalTrials.gov ID; NCT05350969) to assess the efficacy and safety of this drug in patients with reduced LVEF (< 45%) after myocardial infarction. LNP-encapsulated mRNA-0184, which encodes relaxin-2, is in phase I of clinical testing (ClinicalTrials.gov ID; NCT05659264) to assess safety and tolerability of this drug in patients with chronic HF. Both therapies aim to counteract adverse cardiac remodelling, demonstrating the potential of RNA-based treatments in addressing myocardial fibrosis.

### Targeting Oxidative Stress

Oxidative stress results from an imbalance between the production of ROS and the scavenge ability of antioxidants [120]. Modulation of ROS production or the antioxidant defence mechanism may be a promising treatment strategy to minimise oxidative stress and thereby the progression of HF.

Mitochondria are the primary energy producers of the cell, but also contribute to ROS generation and cell death [121]. Modulating the expression of genes involved in mitochondrial metabolism to reduce ROS production may be a therapeutic approach to attenuate the progression of HF. *In vivo,* mitochondria derived from cardiomyocytes were enriched for miR-696, miR-690, and miR-345-3p in the early stages of HF. These miRNAs have been linked to energy metabolism and the oxidative stress pathway [122]. For example, miR-696 negatively regulates the translation of peroxisome proliferator-activated receptor-gamma coactivator (PGC)−1α which stimulates mitochondrial biogenesis and regulates ROS metabolism [123, 124]. Another study found that miR-762 was upregulated and translocated into the mitochondria of cardiomyocytes after anoxia and reoxygenation treatment. It was shown that miR-762 can modulate mitochondrial functions by inhibiting ATP production and complex I enzyme activity, as well as promoting ROS generation and apoptotic cell death. Knockdown of miR-762 subsequently reversed these effects [125].

ROS production is also mediated by enzymes such as by nicotinamide adenine dinucleotide phosphate (NADPH) oxidases and xanthine oxidases (XO) [120]. Increased NADPH oxidase activity has been observed in human failing hearts [126]. *In vitro* inhibition of NADPH oxidase reduced cardiomyocyte apoptosis [127]. This was also confirmed in an *in vivo* rabbit model of myocardial infarction, where reduced oxidative stress, and improved LV dilatation and dysfunction was observed after NADPH oxidase inhibitor treatment [128]. NADPH oxidase with the catalytic subunit NADPH oxidase 2 (NOX2) generates cardiac superoxide (O_2_^−^) [129]. *In vivo* knockdown of NOX2 in mice downregulated NOX2 gene expression and restored fractional shortening [130]. Increased expression and activity of XO has also been observed in patients with HF, and inhibition of this enzyme using oxypurinol and allopurinol improved both cardiac contractility and ejection fraction [131, 132]. Similarly, *in vitro* exposure to hypoxia and reoxygenation, when treated with an XO inhibitor or subjected to XO knockdown, exhibited reduced XO activity and generation of XO-derived products, leading to decreased apoptosis of cardiac cells [133]. Together, inhibiting or silencing ROS-producing enzymes may be a promising treatment strategy for HF.

Inhibition of ROS production can also be achieved by increasing the expression of endogenous antioxidants such as superoxide dismutase (SOD), which is a key antioxidant in the human body. Several miRNAs have been reported to modulate SOD expression, including miR-1, miR-21, miR-199, and miR-126. miR-1 may be a valuable target for RNA therapeutics as its expression is increased in human failing hearts [134] and is associated with reduced SOD activity. Overexpression of miR-1 in cardiomyocytes increases ROS levels and reduces resistance to oxidative stress. Subsequently, co-transfection with anti-miR-1 ASOs effectively reversed the inhibitory effects of miR-1 on SOD activity [135]. Here, redox proteins (i.e. SOD1, GCLC and G6PD) were identified as targets of miR-1. The inhibition of their expression potentially contributes to the elevated ROS levels and increased susceptibility to oxidative stress. Similarly, miR-21 and miR-199 have been shown to negatively regulate SOD expression and thereby contributing to oxidative stress [136, 137]. In contrast, miR-126 overexpression increased SOD activity and reduced ROS levels, which was reversed by miR-126 inhibitory treatment [138]. These findings highlight the potential for modulating specific miRNAs, depending on their different roles, to enhance SOD activity and mitigate ROS production.

### Targeting Inflammation

Chronic exposure to inflammatory cytokines has been observed in human failing hearts [139]. Inflammatory signalling pathways, pro-inflammatory cytokines, or chemokines may serve as a therapeutic target to diminish inflammation and improve cardiac function.

Tumour necrosis factor-α (TNF-α), interleukin (IL)−1, and IL-6 are widely recognized as pro-inflammatory cytokines contributing to the pathogenesis of HF [140–144]. TNF-α is present in human failing hearts, whereas they are absent in non-failing hearts, suggesting that the heart may produce TNF-α during HF [140]. Therefore, targeting TNF-α biosynthesis at the mRNA level may be an interesting treatment strategy. However, previous studies have shown that IL-1 is significantly more potent than TNF-α in inducing an inflammatory response in human and rat cardiac fibroblasts [145–147], positioning IL-1 an interesting therapeutic target. Pharmacological inhibitors of IL-1, such as canakinumab and anakinra, are clinically available and have been shown to reduce recurrent cardiovascular events and hospitalisation for HF, and to improve vascular and LV function [148–150]. However, canakinumab treatment is linked to a higher risk of fatal infection and sepsis than placebo. Thus, alternative therapies without serious adverse events are needed [149]. Modulation of miRNAs that regulate the expression of pro-inflammatory cytokines may be a potential therapeutic target. miR-146, miR-216a, and miR-103 have been shown to reduce the expression of IL-1 and/or TNF-α, whereas miR-128 and miR-145 should be silenced to reduce their expression [151–156]. IL-6 expression may also be affected by modulation of these miRNAs as both TNF-α, and IL-1 modulate IL-6 expression [157, 158]. Lastly, pharmacological inhibition of Nod-like receptor protein 3 (NLRP3), a transcription factor predominantly expressed by proinflammatory macrophages, was able to alleviate right ventricular dilation and dysfunction in an arrhythmogenic mouse model [159].

Pro-inflammatory cytokines, including TNF-α, IL-1, and IL-6, activate the nuclear factor-kappa beta (NF-kB) signalling pathway [160]. In turn, NF-kB promotes the expression of these pro-inflammatory cytokines, creating a positive feedback loop. In addition to cytokines, miRNAs also regulate NF-kB signalling. miR-146 suppresses NF-kB activity, while miR-155 represses a transcription factor that attenuates NF-kB signalling [161, 162]. Subunits of the NF-kB complex are also targeted by miRNAs to influence downstream signalling. For example, miR-145 and miR-329 regulate the NF-kB subunit p65, thereby modulating the expression of TNF-α and IL-6, respectively [163]. These findings suggest that modulation of miRNAs could inhibit NF-kB signalling and potentially reduce inflammation.

Chronic exposure to pro-inflammatory cytokines in HF patients is also mediated by chemokines [164]. Among these, the role of the chemokine C–C Motif Chemokine Ligand 2 (CCL2) in HF has been extensively studied. Upregulation of CCL2 has been demonstrated in experimental models of HF as well as in human failing hearts [165–169]. Therefore, silencing CCL2 may be a potential treatment strategy to reduce inflammation in HF. Previous studies have focused on targeting the interaction of CCL2 with the C–C Motif Chemokine Receptor 2 (CCR2). In an *in vivo* model for autoimmune myocarditis in mice, which were either genetically deficient in CCR2 or treated with siRNA targeting CCR2, reduced inflammation with preserved LVEF was observed compared to control [170, 171]. This suggests that chemokines could also be targeted at the molecular level to minimise inflammation in HF.

An alternative strategy to reduce inflammation is to stimulate anti-inflammatory cytokines by modulating miRNAs. For example, miR-21 modulates the anti-inflammatory cytokine IL-10 by targeting its mRNA, and silencing of miR-21 resulted in a decrease in IL-10 levels [172, 173]. In addition, miR-17–92 and miR-126 are also known to increase IL-10 expression [174, 175]. Overexpression of miR-126 also reduced levels of TNF-α and IL-6, whereas inhibition of miR-126 had the opposite effect [174]. These findings highlight miRNAs as potential therapeutic targets for the modulation of inflammation.

### Targeting Genetic Predisposition and Signal Transduction Leading to Heart Failure

The potential of RNA therapeutics is currently being explored in relation to signalling that leads to HF, for example due to genetic predisposition (e.g. amyloidosis, muscular dystrophy or the pathogenic variant PLN R14del). Amyloidosis is a condition, which can be inherited or acquired, in which misfolded amyloid deposits of transthyretin accumulate in tissues and organs, including the heart, interfering with normal function. Vutrisiran, inotersen, patisiran are RNA therapeutics that target transthyretin for degradation and subsequently halt the progression of amyloidosis [43, 47, 176]. Muscular dystrophy can lead to HF through its effect on the heart. Muscular dystrophies are a group of genetic disorders characterized by progressive muscle weakness and degeneration, caused by defective dystrophin. Casimersen, eteplirsen and golodirsen are RNA therapeutics that promote exon skipping in dystrophin, thereby halting the progression of muscular dystrophy [18, 45, 177]. The potential of RNA therapeutics is currently being investigated to treat genetic forms of HF such as PLN R14del [40]. In this disease, antisense inhibition of PLN attenuated HF in an in vivo mouse model [55, 178]. RNA therapeutics can be tailored to target the signaling pathways that lead to HF, and have great potential in the treatment of genetic forms of HF. Beyond genetic predisposition, signal transduction routes such as the G protein-coupled receptor kinases (GRK) or endothelial dysfunction can contribute to HF. The GRK interactome, particularly GRK2 and GRK5, contribute to HF by modulating β-adrenergic signaling, inflammatory pathways and cardiac remodeling. RNA therapeutics offer a promising strategy to selectively downregulate GRK expression or disrupt pathological interactions. For example, miRNA-133a has been identified as a potential regulator of GRK. This targeted modulation may restore receptor sensitivity and improve cardiac function [179]. In addition, endothelial dysfunction contributes to HF progression by impairing vasodilatory signaling and promoting inflammation, via previously described pathways such as TGF-β and NF-kB. These signaling cascades reduce nitric oxide bioavailability, increase vascular permeability, and promote leukocyte adhesion. miR-21 and miR-92 are key regulators of endothelial function and implicated in HF, and modulated by SGLT2 inhibitors [180]. RNA therapeutics targeting endothelial regulators offer a novel approach to restore vascular integrity and reduce cardiac stress.

## Discussion

The complexity of HF results from several interrelated pathophysiological processes. Traditional therapeutic strategies often focus on symptomatic relief but fail to address the underlying mechanisms of the disease. This review highlights the potential of RNA therapeutics, including RNA interference and RNA-enhancing strategies, as transformative tools to target key hallmarks of HF, including dysregulated calcium handling, myocardial fibrosis, oxidative stress, inflammation, and genetic predisposition. By harnessing the specificity and versatility of RNA therapeutics, it is possible to modulate gene expression and restore molecular physiology in the failing heart. Here, RNA interference therapies, such as siRNAs and miRNAs, allow precise silencing of pathological genes, while RNA-enhancing modalities, such as mRNA therapies, allow the restoration or enhancement of beneficial protein functions. Advances in delivery systems, such as lipid nanoparticles and cardiac-targeted conjugates, are overcoming many of the challenges associated with the stability and off-target effects of RNA therapeutics, bringing them closer to clinical application. Promising molecular targets for RNA therapeutics allow us to modulate hallmarks of HF including calcium handling, myocardial fibrosis, oxidative stress, and genetic predisposition, to restore physiology in the failing heart (Table 1).

**Table 1:** Therapeutics targets for RNA therapeutics in heart failure

Hallmark of Heart Failure	Cellular Process	Therapeutic Target	Model	Outcome	Ref
Calcium Handling	Calcium Exchangers	CaV1.2, RyR2, NCX	*In vivo*, *in vitro*	Improved Ca^2^⁺ handling, arrhythmia reduction	[56–62]
	Calcium-related kinases/phosphatases	CaMKII, PKA, PP1	*In vivo*, clinical ongoing	Reduced remodeling, improved contractility	[63–69]
	Calcium Regulatory Proteins	SERCA2a, PLN, DWORF	*In vivo*, *In vitro*, clinical (SERCA2a)	Enhanced contractility, HF symptom reduction	[55, 70–78]
	Calcium-related miRNAs	miR-1, miR-25, miR-133	*In vivo*, *in vitro*	Improved function, reduced fibrosis and apoptosis	[79–85]
	Calcium Sensitizers	Troponin C	*In vitro*	Altered myofilament sensitivity	[86, 87]
Myocardial Fibrosis	Fibroblast to Myofibroblast Transition	miR-21, miR-200, miR-519d	*In vivo*	Promoted or attenuated FMT	[92–99]
	Collagen Synthesis	COL1A1, COL3A1	*In vitro*	Reduced myocardial collagen synthesis, and fibrosis	[111, 112]
	Fibroblast Activation	TGF-β, CTGF, miR-181c	*In vivo*, *In vitro* (miR-181c)	Attenuated fibrosis, preserved function	[90, 100–106]
	Extracellular Matrix Remodeling	MMPs, TIMPs	*In vivo*, *in vitro*	Improved contractility	[113–119]
Oxidative Stress	ROS-Producing Enzymes	NOX2. XO	*In vivo*, *in vitro*	Decreased ROS and apoptosis, improved function	[126–133]
	Antioxidant Enzymes	SOD	*In vivo*	Reduced oxidative stress, preserved function	[134–138]
	Mitochondrial Dysfunction	miR-696, 690, 345	Not applicable	Unexplored in HF	[122, 123]
Inflammation	Pro-Inflammatory Cytokines	TNF-α, IL-6, IL-1	*In vivo*, clinical (IL-1)	Reduced inflammation, improved remodeling	[140–158]
	Inflammatory Signaling	NF-κB	*In vivo*	Suppressed cytokine production	[160–163]
	Anti-Inflammatory Cytokines	IL-10	*In vivo*	Protective effect, reduced fibrosis	[172–175]
	Inflammatory Mediators	CCL2	*In vivo*	Decreased immune infiltration	[164–171]
Genetic predisposition	Amyloidosis	Transthyretin	Clinical	Stabilized protein, reduced cardiotoxicity	[43, 47, 176]
	Muscular Dystrophy	Dystrophin	*In vivo*, clinical	Restored protein expression, improved function	[18, 45, 177]
	Pathogenic Variants	PLN R14del	*In vitro*, in vivo	Reduced PLN, attenuated HF	[55, 178]
HF-related signal transduction	GRK interactome, GRK2, GRK5	miRNA-133a	*In vivo*	Diminished cell death, preserved cardiac function	[179]
	Endothelial dysfunction	miR-21, miR-92	Clinical	Upregulated in HF, restored after empagliflozin	[180]

The potential of RNA therapeutics to treat the multifaceted nature of HF represents the future of the treatment of HF. Future research should focus on refining delivery mechanisms, minimizing immunogenicity, and conducting clinical trials to translate these therapeutic targets into effective treatments. With continued innovation and multidisciplinary collaboration, RNA therapeutics hold the promise of addressing unmet needs in HF and improving outcomes for patients with HF.

## Data Availability

Not applicable.

## Abbreviations

*α-SMA*: Alfa smooth muscle actin
*ASOs*: Antisense oligonucleotides
*Ca*^*2+*^: Calcium
*CaMKII*: Ca^2+^/calmodulin-dependent protein kinase II
*Cav1.2*: L-type Ca^2+^ channel
*CCL2*: C-C Motif Chemokine Ligand 2
*CCR2*: C-C Motif Chemokine Receptor 2
*circRNA*: Circular RNA
*COL1A1*: Collagen type l alpha
*CTGF*: Connective tissue growth factor
*DWORF*: Dwarf open reading frame
*ECM*: Extracellular matrix
*FMT*: Fibroblast to myofibroblast transition
*GRK*: G-protein coupled receptors kinases
*HF*: Heart failure
*IL*: Interleukin
*lncRNAs*: Long non-coding RNAs
*LVEF*: Left ventricular ejection fraction
*MMPs*: Matrix metalloproteinases
*miRNA*: MicroRNA
*mRNAs*: Messenger RNAs
*NADPH*: Nicotinamide adenine dinucleotide phosphate
*NCX*: Sodium/Ca^2+^ Exchanger
*NF-kB*: Nuclear factor-kappa beta
*NLRP3*: Nod-like receptor protein 3
*NOS*: Nitric oxide synthase
*NOX2*: Nicotinamide adenine dinucleotide phosphate oxidase 2
*O*_*2*_^*−*^: Superoxide
*PKA*: Protein kinase A
*PLN*: Phospholamban
*PP1*: Protein phosphatase 1
*RISC*: RNA-induced silencing complex
*RNAi*: RNA interference
*RNAse H*: Ribonuclease H
*ROS*: Reactive oxygen species
*RyR2*: Ryanodine receptors
*saRNAs*: Small activating RNAs
*SERCA2a*: Sarcoplasmic reticulum Ca^2+^ ATPase 2a
*shRNA*: Short hairpin
*siRNAs*: Small interfering RNAs
*SIRT7*: Sirtuin 7
*SOD*: Superoxide dismutase
*ss-siRNA*: Single-stranded siRNA
*TAB3*: TGF-β-activated kinase 1
*TGF-β*: Tissue growth factor-β
*TGF-βR1*: TGF-β receptor type 1
*TIMPs*: Matrix metalloproteinases
*TNF-α*: Tumour necrosis factor alfa
*XO*: Xanthine oxidases
